# Effect of various dialysis modalities on intradialytic hemodynamics, tissue injury and patient discomfort in chronic dialysis patients: design of a randomized cross-over study (HOLLANT)

**DOI:** 10.1186/s12882-021-02331-z

**Published:** 2021-04-15

**Authors:** Paul A. Rootjes, Menso J. Nubé, Camiel L. M. de Roij van Zuijdewijn, Gertrude Wijngaarden, Muriel P. C. Grooteman

**Affiliations:** Department of Nephrology and Amsterdam Cardiovascular Sciences (ACS), Amsterdam UMC, VU University of Amsterdam, Amsterdam, The Netherlands

**Keywords:** Hemodialysis, Hemodiafiltration, Intradialytic blood pressure, Intradialytic tissue injury, Intradialytic patient tolerance, Multicentre randomized controlled cross-over trial

## Abstract

**Background:**

From a recent meta-analysis it appeared that online post-dilution hemodiafiltration (HDF), especially with a high convection volume (HV-HDF), is associated with superior overall and cardiovascular survival, if compared to standard hemodialysis (HD). The mechanism(s) behind this effect, however, is (are) still unclear. In this respect, a lower incidence of intradialytic hypotension (IDH), and hence less tissue injury, may play a role. To address these items, the HOLLANT study was designed.

**Methods:**

HOLLANT is a Dutch multicentre randomized controlled cross-over trial. In total, 40 prevalent dialysis patients will be included and, after a run-in phase, exposed to standard HD, HD with cooled dialysate, low-volume HDF and high-volume HDF (Dialog iQ® machine) in a randomized fashion. The primary endpoint is an intradialytic nadir in systolic blood pressure (SBP) of < 90 and < 100 mmHg for patients with predialysis SBP < 159 and ≥ 160 mmHg, respectively. The main secondary outcomes are 1) intradialytic left ventricle (LV) chamber quantification and deformation, 2) intradialytic hemodynamic profile of SBP, diastolic blood pressure (DBP), mean arterial pressure (MAP) and pulse pressure (PP), 3) organ and tissue damage, such as the release of specific cellular components, and 4) patient reported symptoms and thermal perceptions during each modality.

**Discussion:**

The current trial is primarily designed to test the hypothesis that a lower incidence of intradialytic hypotension contributes to the superior survival of (HV)-HDF. A secondary objective of this investigation is the question whether changes in the intradialytic blood pressure profile correlate with organ dysfunction and tissue damage, and/or patient discomfort.

**Trial registration:**

Registered Report Identifier: NCT03249532# (ClinicalTrials.gov). Date of registration: 2017/08/15.

## Background

Despite the use of high permeable dialyzers, which combine diffusive with convective transport, the clinical outcome of hemodialysis (HD) patients remains poor. In online post-dilution hemodiafiltration (HDF), diffusion is by and large similar to HD, while the amount of convective transport is considerable higher. Recently, four randomized controlled trials (RCT) have been published which compared HD with HDF [1–4]. Although the results of the individual studies were inconclusive, a recent meta-analysis on individual participant data (IPD) showed a superior outcome for patients treated with HDF (all-cause mortality HR 0.86 [95% CI: 0.75; 0.99]). The largest mortality reduction was obtained in patients receiving the highest convection volume (high-volume HDF [HV-HDF] > 23 L/1.73 m^2^/session: all-cause mortality HR 0.78 [95% CI: 0.62; 0.98], if compared to HD) [5].

Nonetheless, it is still unclear why HV-HDF is associated with an improved survival [6]. On theoretical grounds, both the enhanced removal of middle molecular weight uremic retention products and a superior bio-incompatibility (BI) profile [7], including less inflammation [8, 9] and less dialysis-induced hypoxia [10, 11], may play an important role. Moreover, since treatment with HDF has been associated with a lower incidence of intradialytic hypotension (IDH) than standard HD [12–15], in which an altered sodium removal during HDF may play a role [16], a lesser amount of dialysis-induced tissue damage may also contribute to the beneficial effect of HDF on survival.

Since IDH is a frequently occurring side-effect of HD treatment and microcirculatory dysfunction is a prominent feature of patients with advanced chronic kidney disease (CKD) [17, 18], every single dialysis session may further deteriorate the already affected structure and function of vital organs, such as the brain, gut and heart [19]. After all, HD-associated cardiomyopathy may result not only from the various inflammatory and metabolic derangements of pre-dialysis CKD [20], but also from HD-induced perfusion deficits [21–27], which is considered a model of repetitive organ ischemia-reperfusion injury. In this respect it is interesting to note that an absolute intradialytic nadir of SBP < 90 and < 100 mmHg for patients with a predialysis SBP < 159 and ≥ 160 mmHg, respectively, appeared most strongly associated with mortality in a large study, comparing the relation between various definitions of IDH and outcome [28]. Considering the gut, HD-induced hypoperfusion may cause disruption of the intestinal barrier and permit the translocation of gut-derived endotoxins, bacterial DNA and/or intact bacteria into the blood. Circulating bacterial fragments may contribute to systemic inflammation, cardiovascular disease, and reduced survival in these patients [29–34].

As intradialytic blood pressure measurements were not the primary endpoint in any of the above-mentioned studies in HDF patients [12–15], it is still unclear if, and if so, *why* this modality is associated with less IDH than standard HD (S-HD). Since IDH can be alleviated by HD treatment with cooled dialysate (C-HD) and (HV-) HDF may induce cooling of the extracorporeal circuit and subsequent cooling of the patient‚ thermal factors may play an important role in this respect [12–15, 35–44] and are even considered to be exclusively responsible for the superior outcome of HDF treatment [45]. Yet, despite the physical benefits of a reduction in IDH and related symptoms, patients may suffer from shivering and cold (sensations). Interestingly, from a large recent RCT it appeared that intradialytic patient tolerance was significantly better during HDF, if compared to treatment with S-HD [3]. As of yet, however, it is unknown which intermittent extracorporeal renal replacement therapy (S-HD, C-HD, LV-HDF or HV-HDF) has the most favorable intradialytic patient tolerance profile. Altogether, current data suggests that (HV)-HDF is the preferred treatment to circumvent dialysis-induced IDH, and hence, to alleviate the repetitive microcirculatory dysfunction and subsequent tissue damage of dialysis treatment. So far, however, comparative data between S-HD, C-HD, LV-HDF and HV-HDF are lacking.

Therefore, the protocol for the current investigation with the original title “Effect of high-volume Online hemodiafiLtration on intradiaLytic hemodynamic (iN)sTability and cardiac function in chronic hemodialysis patients” (HOLLANT) was designed. In this randomized cross-over clinical study, not only intradialytic hemodynamics will be investigated, but also changes in cardiac performance and signs of myocardial injury. In addition, besides markers of inflammation and oxidative stress, intradialytic tissue damage, as indicated by various cell surface markers and the transfer of microbial DNA (mDNA) from the gut to the blood, will be investigated. Hence, finally, since cool dialysate may induce cold (sensations) and shivering [37], patient tolerance and thermal perception will be compared between the four modalities.

### Objectives

The primary objective of the HOLLANT study is to evaluate whether intradialytic hemodynamic stability is better preserved during HV-HDF, by comparing the frequency of intradialytic hypotensive episodes between S-HD, C-HD, LV-HDF and HV-HDF. The main secondary objectives include intradialytic signs of tissue damage as measured by Speckle Tracking Echocardiography (STE), a diversity of laboratory parameters, and patient reported outcome measures (PROMs), such as tolerance and cold (sensations).

## Methods

### Study design

The HOLLANT study is an open, cross-over, multicenter, intervention RCT in chronic intermittent dialysis patients who will be exposed to four different dialysis modalities: 1) S-HD: HD with a dialysate temperature of 36.5 °C. 2) C-HD: HD with a dialysate temperature 35.5 °C. 3) LV-HDF: HDF with a convection volume of 15 L/1.73 m^2^/session and a dialysate temperature of 36.5 °C. 4) HV-HDF: HDF with a convection volume of ≥23 L/1.73 m^2^/session and a dialysate temperature of 36.5 °C.

**Fig. 1:**
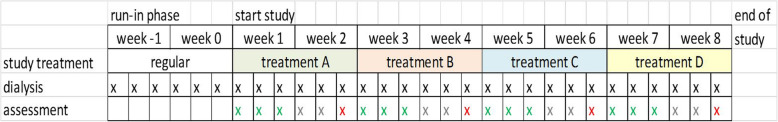
Overview of study scheme. The run-in phase during week − 1 and week 0 (total duration of 2 weeks) is followed by the four different dialysis treatment modalities (S-HD, C-HD, LV-HDF, HV-HDF) in a randomized order (total duration of 8 weeks).  = Non-invasive advanced hemodynamic monitoring (Clearsight) during the first or second treatment of the last week and measurement of blood pressure every 15 min;  = assessment of STE, blood sampling and measurement of blood pressure every 15 min;  = measurement of blood pressure every 30 min

After enrollment, participants will be randomized centrally to the four dialysis modalities by using computer randomization software [46]. Randomization occurs in blocks. Enrolled patients will be treated with their usual dialysis modality (either HD or HDF) during the first 2 weeks of the study. Patients who cannot achieve or tolerate a blood flow rate ≥ 350 mL/min during this run-in phase will be excluded before the actual start of the study. Treatment times will be fixed at 4 h per session during the entire conduct of the study. Thereafter, patients start the study-phase and will be treated with S-HD, C-HD, LV-HDF or HV-HDF in a random order. Each dialysis modality will last 2 weeks. The total study duration is 2 (run-in phase) + 8 (study phase) = 10 weeks per patient. An overview of the study is shown in Fig. 1.

### Study population

We plan to include 40 patients. For reasoning (see “Sample size considerations” below), patients will be recruited from three dialysis facilities: a commercial dialysis clinic (Niercentrum aan de Amstel, Amstelveen, The Netherlands), a large community based clinical hospital (St. Antonius Hospital, Nieuwegein, The Netherlands) and a university hospital (Amsterdam UMC, location VU University Medical Center, Amsterdam, The Netherlands). The in- and exclusion criteria are depicted in Table 1. Severe incompliance to the dialysis procedure and accompanying prescriptions is defined as non-adherence to the dialysis prescription.

**Table 1:** Inclusion and exclusion criteria

**Inclusion criteria**	
- Patients treated with HD or HDF 3 x per week during at least 4 h for at least 2 months.	
- Ability to understand study procedures.	
- Willingness to provide informed consent.	
- Dialysis single-pool Kt/V for urea (spKt/Vurea) ≥ 1.2^a^	
- Achievement of a blood flow of ≥350 ml/min and/or convection volume of ≥23 Liter per treatment during the run-in phase.	
- Dialysis access recirculation < 10%^a^.	
**Exclusion criteria**	
- Age < 18 years.	
- Life expectancy < 3 months.	
- Participation in another clinical intervention trial.	
- Severe incompliance to the dialysis procedure and accompanying prescriptions.	

^a^Based on the most recent (before start of the study) measurements from daily practice, in accordance with the applicable national and international guidelines [47, 48]

### Dialysis prescription and equipment

#### Dialysis modalities

HDF will be performed in the post-dilution mode with a target convection volume (substitution volume + net ultrafiltration [UF] volume) of 15 L (LV-HDF) or 25 L (≥23 L, HV-HDF). Extracorporeal blood flow rate will be targeted at 350–400 ml/min and filtration fraction (blood flow rate / convection flow rate) at 25–30%, which have been proven to be feasible [49]. Substitution fluid is prepared from the dialysis fluid by one additional step of UF with a dialysis fluid filter (Diacap® Ultra, B. Braun Avitum AG, Melsungen, Germany), before it is infused into the blood after the dialyzer. The electrolyte composition of the substitution fluid is identical to the electrolyte composition of the dialysate.

For a given patient, treatment settings will be kept similar in all treatment modalities, e.g. UF profile, start of treatment with either empty or filled lines, blood flow rate, session length and type of anticoagulant. Any clinically necessary change will be documented.

#### Dialyzers

Both HD and HDF will be performed with high-flux dialyzers: Xevonta® 23 dialyzers (membrane material: Amembris, i.e. polysulfon-based membrane with polyvinylpyrrolidone; UF coefficient: 124 ml/min/mmHg; surface area 2.3 m^2^; sterilization by gamma radiation; capillary internal diameter 195 μm; B. Braun Avitum AG, Melsungen, Germany). In exceptional cases, the attending nephrologist can decide to treat the patient with a Xevonta® 18 dialyzer (membrane material: Amembris, UF coefficient 99 ml/min/mmHg; surface area 1.8 m^2^; sterilization by gamma radiation; capillary internal diameter 195 μm; B. Braun, Avitum AG, Melsungen, Germany), or comparable.

#### Dialysis machines

All dialysis treatments will be performed on the Dialog iQ® dialysis machine equipped with Adimea®, automatic blood pressure monitor (ABPM), Hematocrit (HCT) sensor with integrated oxygen saturation (spO2) monitoring device, mantled with the captive tubing system DiaStream® iQ (all B. Braun Avitum AG, Melsungen, Germany).

#### Dialysis fluids

All treatments will be performed with ultrapure (UP) dialysis fluids (< 0.1 CFU [colony forming units]/ml, < 0.03 EU [endotoxin units]/ml). Dialysate flow rate will be 500 mL/min during S-HD and C-HD, and 600 ml/min in LV- and HV-HDF (as the substitution fluid is derived from the dialysate flow, the dialysis machine automatically increases the dialysate flow during online HDF). The electrolyte composition of the dialysis fluid is: Na 138–140 mmol/L; K 2.0–3.0 mmol/L; HCO3 30–35 mmol/L; Ca 1.25–1.75 mmol/L; Mg 0.5 mmol/L; Cl 108–109.5 mmol/L; glucose 5.6 mmol/L; acetate 3 mmol/L and will not be changed for each individual patient during the conduct of the study.

### Patient care

Routine patient care is performed according to current national [47] and international [48] quality of care guidelines, including the measurement of single-pool Kt/V for urea (spKt/Vurea) and access recirculation. As in daily clinical practice, dry-weight is assessed weekly by the attending nephrologist by evaluating clinical symptoms, edema and blood pressure before, during and after dialysis. All patients will be administered their usual dose of low molecular weight heparin (LMWH) anticoagulation (i.e. nadroparin or dalteparin). No sodium profiling will be applied and the conductivity of the dialysis fluid will be recorded continuously by the Dialog iQ dialysis machine during the conduct of the study. When a symptomatic IDH occurs during treatment with a dialysate temperature (Td) of 36.5 °C and there is no reaction to fluid administration, Td will be lowered by 0.5–1.0 °C according to standard protocol and noted on patients’ individual record list.

### Data collection

Table 2 provides an overview of the data that will be collected during the study.

**Table 2:** Overview of collected variables

*Hemodynamic parameters*	Intradialytic SBP, DBP, MAP, PP, HR, IDH events, Clearsight measurements
*Markers of cardiac damage*	CK-MB, EVs, intradialytic speckle tracking echocardiography
*Markers of endothelial damage*	sv-ICAM-1, EVs
*Markers of gut ischemia*	mDNA, sCD14
*Markers of inflammation*	hs-CRP, IL-6R, EVs
*Special biomarkers*	FGF23 C-term, EVs
*Patient tolerance*	DSI, VAS-TP
*Others*	pO2 from the arterial line, SaO2, body temperature

#### Baseline data registration

At baseline, all relevant information will be documented: i.e. demographical data, information on cardiovascular disease (CVD), cause of renal failure, time on dialysis, co-morbidity, medical history and medication. A history of CVD is defined as a confirmative answer on any of the questions regarding a previous acute myocardial infarction, coronary artery bypass graft, percutaneous transluminal coronary angioplasty, angina pectoris, stroke, transient ischemic attack, intermittent claudication, amputation, percutaneous transluminal angioplasty, peripheral bypass surgery, and renal percutaneous transluminal angioplasty.

#### Baseline laboratory assessments

Data will be used from the last routine assessment as indicated by the national guideline [47]: Hb, Ht, phosphate, calcium, parathyroid hormone (PTH); spKt/V urea (monthly). Residual kidney function will be expressed as the estimated GFR (eGFR), calculated by the mean of 24-h urinary creatinine and urea clearances and adjusted for body surface area (mL/min/1.73 m2). The plasma concentrations used for this calculation are the mean of the values before and after dialysis. eGFR is considered zero in patients with a urinary production < 100 mL/day.

#### Dialysis-related recordings

Before dialysis, the bodyweight and the interdialytic weight gain (IDWG) of the patients will be noted. During dialysis, hemodynamics will be recorded according to the protocol (see below) as well as all other treatment related variables, including UF volume, UF rate and online monitoring of estimated Kt/V from UV-absorbance measurements in spent dialysate via Adimea® [50]. Furthermore, data on the anticoagulation type and dose, vascular access (central venous catheter [CVC], graft or AV fistula, including documentation of distance between arterial needle and anastomosis in the 2 latter, vascular access flow (if fistula or graft), needle size and type, blood pump speed, dialysis machine and dialyzer. In case of HDF treatment, the achieved convection volume will be noted at the end of each procedure. Convection volumes will be calculated as the sum of the intradialytic weight loss and the substitution volume in L/session. Finally, at the end of each dialysis procedure the patients achieved dry weight will be recorded.

#### Patient tolerance

**Fig. 2:**
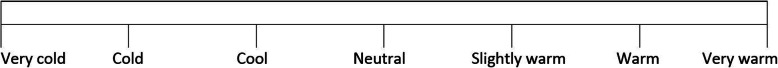
Visual Analogue Scale – Thermal Perception (VAS-TP). Thermal perception will be assessed by the Visual Analogue Scale - Thermal Perception (VAS-TP) before HD(F) and after 1 and 3 h. Upon the continuous line patients can indicate their actual thermal perception

Since simple questions on intradialytic symptoms appear to predict patient outcomes better than complicated questionnaires [51–53], a modified version of the Dialysis Symptom Index (DSI) will be handed out after each treatment period (Table 3). Thermal perception will be assessed by the Visual Analogue Scale - Thermal Perception (VAS-TP) [54, 55], before HD(F) and after 1 and 3 h (Fig. 2). Upon a continuous line, patients can indicate their actual thermal perception.

**Table 3:** Modified Dialysis Symptom Index (DSI)

During the past week: did you experience this symptom?	Not at all	A little bit	Some-what	Quite a bit	Very much
1. Dizziness or lightheadedness	0	1	2	3	4
2. Nausea	0	1	2	3	4
3. Vomiting	0	1	2	3	4
4. Headache	0	1	2	3	4
5. Muscle cramps	0	1	2	3	4
6. Swelling of the legs	0	1	2	3	4
7. Shortness of breath	0	1	2	3	4
8. Chest pain	0	1	2	3	4
9. Itching	0	1	2	3	4
10. Feeling cold	0	1	2	3	4
11. Shivering	0	1	2	3	4
12. Feeling tired or lack of energy	0	1	2	3	4
13. Recovery time after dialysis: 0 = none 1 = after 1 h 2 = after half a day 3 = the next day 4 = the day of the next dialysis	0	1	2	3	4
Are there any other symptoms not mentioned on this questionnaire that you have experienced during the past week?

#### Hemodynamic monitoring

Diastolic (DBP) and systolic (SBP) blood pressure, pulse pressure (SBP minus DBP), mean arterial pressure (MAP: [(SBP + 2*DBP)/3]), heart rate (HR) and intradialytic hypotensive episodes (IDH, see Primary endpoint) will be recorded before, after and during each dialysis treatment every 30 min (during the first week of every treatment modality) and every 15 min (during the second week). Measurements will be performed with a manometric cuff in different sizes, which is connected to the dialysis machine (automatic blood pressure measurement cuff, B. Braun Avitum AG, Melsungen, Germany).

#### Non-invasive advanced hemodynamic monitoring

During the first or second dialysis of the last week of every treatment modality, every 20 seconds MAP, stroke volume (SV), heart rate, total peripheral resistance and cardiac output (CO) will be obtained using the Clearsight monitor (BMEYE/Edwards Lifesciences, Amsterdam, The Netherlands), which is a non-invasive arterial pressure measuring device. This monitor turns finger arterial pressure with a fast-pneumatic system to account, as well as photoplethysmography to detect changes in finger arterial diameter during inflations [56]. A volume clamp method is used whereby rapid variations in the cuff pressure allow maintenance of a constant arterial diameter, with an automatic algorithm (Physiocal). The pressure within the cuff is therefore reflective of finger arterial pressure [56]. The Clearsight finger cuff will be attached to the mid-phalanx of the third digit of the hand at the contralateral side of the dialysis shunt. The heart reference system will be placed on the middle of the left side of the thorax. The measured hand and the manometric arm cuff are placed at the mid-thorax to account for hydrostatic pressure differences.

#### Body temperature

Body temperature will be assessed before and after each dialysis session by a tympanic thermometer (Genius 2 Tympanic Thermometer, Covidien, Mansfield, USA).

#### 2D speckle tracking echocardiography

Speckle Tracking Echocardiography (STE) will be performed before, after 1 and after 3 h of treatment during the last dialysis of each modality. Two-dimensional left ventricular (LV) measurements of wall thickness and cavity diameters, 2D biplane Simpson measurement of volumes and ejection fraction are measured using a commercial scanner (Affiniti 70C, Philips Healthcare, The Netherlands) with a fully sampled matrix array (S5–1) transducer. These measurements are indexed for body surface area (BSA). For the assessment of global longitudinal deformation (GLS) as well as distribution of regional longitudinal strain, 2DSTE is performed from an apical position. Diastolic LV function is measured by 2D LAVI (left atrial volume index), transmitral E/A ratio and deceleration time, e’ wave velocity, E/e’ ratio and SPAP (systolic pulmonary artery pressure). Estimated right ventricular function will be done by TAPSE (tricuspid annular plane systolic excursion), s’ wave velocity and RV (right ventricular) regional and global strain. The data will be recorded electronically and assessed off-line by a trained research physician in Xcelera Cardiology Information Management Version 4.1 (Philips Healthcare, The Netherlands).

#### Blood sampling

Blood sampling will be performed in the last session of each dialysis modality, after the long interdialytic interval (see Fig. 1). Blood samples will be drawn from the arterial line of the extracorporeal circuit (ECC) before treatment (but after administration of LMWH) and after 4 h of treatment (see Table 2). After processing, all blood samples will be stored at − 80 °C until assessment in the central laboratory of the hospital of the Amsterdam University Medical Centers.

#### Markers of inflammation, cardiac- and endothelial damage and FGF23

Soluble CD14 (sCD14), interleukin-6 receptor (IL-6R), high sensitive C-reactive protein (hs-CRP), creatine kinase myocardial band (CK-MB), soluble vascular intercellular cell adhesion molecule 1 (sv-ICAM-1), and fibroblast growth factor 23 C-terminal (FGF23 C-term) will be assessed in EDTA or heparin plasma samples after being placed on ice and centrifuged within 30 min, at 1800 g for 10 min.

#### Relative blood volume and oxygenation

Hematocrit measurements, reflecting relative blood volume (RBV), and spO2 will be assessed continuously in the ECC by the HCT sensor and with the integrated spO2-monitoring device located on the Dialog iQ® dialysis machine (B. Braun Avitum AG, Melsungen Germany). RBV- and spO2 values will be read out from the trend-files, which are retrieved by service technicians. In addition, blood samples for pO2, pH, bicarbonate and base excess analysis will be drawn from the arterial line before and after treatment (at the moment of activation and before de-activation of the oxygen saturation monitoring device on the Dialog iQ®), and assessed directly with a point-of-care device (Epoc blood analysis, Epocal Inc., Ottawa, ON, Canada).

#### Bacterial DNA

Microbial DNA (mDNA) will be assessed in EDTA blood with 16S–23S interspace (IS) pro after DNA isolation. In short, IS-pro is a eubacterial PCR-based technique for detection of most bacterial species within a sample, and is based on length and sequence polymorphisms of the bacterial 16S–23S ribosomal interspace regions. IS-pro has been optimized to detect bacterial loads as low as 1 CFU per 5 ml blood [57, 58].

#### Extracellular vesicles

Citrate blood samples will be centrifuged for 15 min at 2500 g, 20 °C to remove red blood cells. Subsequently, the EV-containing supernatant is isolated and centrifuged again (15 min at 2500 g, 20 °C). The EV-containing supernatant will be frozen and stored at − 80 °C until further use. Before assessment, samples will be thawed at 37 °C. Subsequently, direct measurement of the EVs in plasma will take place, as extensively described by van der Pol et al. [59].

### Endpoints

#### Primary endpoint

The primary endpoint of this study is an absolute intradialytic nadir in SBP of < 90 and < 100 mmHg for patients with a predialysis SBP < 159 and ≥ 160, respectively.

#### Secondary endpoints

Secondary endpoints include: 1) intradialytic LV chamber quantification and deformation (longitudinal function with speckle tracking) and LV diastolic function; 2) the intradialytic hemodynamic profile of SBP, DBP, MAP and PP; 3) organ and tissue damage, such as the release of specific cellular components; and 4) patient-reported symptoms and thermal perceptions during each modality.

### Statistical methods

Descriptive data will be represented as mean ± standard deviation (SD), median (interquartile range) or number (percentage), when appropriate. The between-treatment number of IDH episodes (based on an intradialytic nadir in SBP as described above) will be analyzed using regression analysis with a Poisson distribution. The mean inter-treatment variability of continuous variables will be calculated using generalized linear mixed effects models with a random intercept, random slope or both, based on the lowest Aikaike’s Information Criterion. A two-sided *p-*value ≤0.05 is considered statistically significant. Statistical evaluations will be performed using IBM SPSS Statistics version 26.0 (Chicago, IL, USA) or RStudio version 1.1.456.

#### Sample size considerations

We plan to include 40 patients. Loss to follow-up (due to an event) is expected to be about 20%. The number of patients with complete follow-up will be sufficient to detect a 40% lower risk (relative risk of 0.60, α = 0.05, β = 0.80) in the occurrence of the primary endpoint. The power calculation applied was designed for cross-over studies [60].

#### Data management

Clinical data will be extracted from the hospital’s electronic information systems and dialysis machines. Data will be collected on electronic case record forms (CRF) using web-based Castor EDC [61]. Data will be entered in the data file by an experienced research nurse or research physician. Beforehand, ranges will be defined in the electronic file for all data values to ensure their validity and integrity. Data entry will be checked for 5% of randomly selected CRFs. Patient data will be coded. Analyzing and publication of the results of this study will be performed anonymously.

### Potential consequences of the COVID-19 pandemic

Given the current COVID-19 pandemic and the fact that this study is carried out in a vulnerable population, it is conceivable that the practical implementation of this study will encounter some serious obstacles. As such, interventions requiring direct patient contact and performed solely in the interest of the study, such as cardiac ultrasounds, may be undesirable in times of alarming virus spread.

## Discussion

The HOLLANT study is principally designed to evaluate prospectively whether intradialytic hypotension, as defined under the primary endpoint, is less frequently observed during HV-HDF than during the three other modalities (S-HD, C-HD and LV-HDF). Second, the study aims to assess whether a more favourable intradialytic hemodynamic profile, as measured by various hemodynamic parameters including MAP and DBP, is associated with a lower degree of tissue injury and organ dysfunction, and a better patient tolerance. After all, it is conceivable that a lower blood pressure and/or diminished perfusion is accompanied by tissue injury, organ dysfunction and subjective discomfort, since pre-existing microcirculatory dysfunction is already present in the majority of patients with advanced CKD [17]. In the present study, all these aspects are addressed and, depending on the outcome, analysed for potential interrelationships.

As for the heart, functional changes as observed by STE, will be correlated with intradialytic hemodynamics and with biochemical signs of myocardial damage, such as an increase in the blood levels of CK-MB and release of EVs from cardiomyocytes. Considering the gut, hypoperfusion of the intestinal microcirculation may promote enterocyte injury, loss of the gut-blood-barrier function and increased permeability, thus permitting the intradialytic transfer of mDNA into the circulation. Any increase during dialysis will be correlated with both blood pressure alterations and changes in the inflammatory state, as measured by sCD14 and IL-6R. Tissue injury of various origin, including circulating blood cell elements, the endothelium and cardiomyocytes will be assessed by measuring alterations in the blood levels of EVs. The latter parameters will be correlated with intradialytic hemodynamics as well, and the aforementioned signs of tissue injury, including a rise in CK-MB and mDNA in the blood. Finally, all topics will be related to changes in tympanic body temperature, oxygen and inflammatory status, patient reported temperature sensations and treatment tolerance.

If HV-HDF appears indeed to be associated with a better intradialytic hemodynamic profile and less tissue damage, this will enhance our understanding why HDF is associated with a superior patient outcome and allow the nephrological community to improve this treatment modality even further. If not, the outcome of this study will anyhow teach us if, and to what extent intradialytic hemodynamic instability is associated with tissue injury and patient discomfort, and thus help the medical staff to optimize dialysis treatment in individual cases.

The largest strength of this study is its randomized cross-over design, which prevents that inter-individual differences, such as co-morbidity and prescribed medication, influence the results. An important limitation may be the duration of each dialysis modality, which may be too short to answer all questions. Yet, to minimize patients’ discomfort the underlying protocol was designed in its current form. Since this study is primarily designed to investigate the intradialytic hemodynamics and its consequences on organ and tissue damage between the four most practiced extracorporeal dialysis techniques (S-HD, C-HD, LV-HDF and HV-HDF), potential differences in thermal and sodium balance are not addressed. Another important issue of any study with a cross-over design is the duration of the wash-out period that will most likely prevent carry-over effects of the previous modality. For this study, a wash-out period of 1 week (the first week of every treatment modality) was arbitrarily chosen, since relevant information on this topic is absent.

### Trial status

The recruitment and inclusion of patients started at 29-08-2018. Currently, 25 (62.5%) of the intended 40 patients have been included in the study.

## Data Availability

The datasets generated and/or analyzed during the current study are not publicly available, but are available from the corresponding author on reasonable request.

## Abbreviations

BP: Blood pressure
BI: Bio-incompatibility
BSA: Body surface area
CVC: Central venous catheter
CRF: Case record forms
CK-MB: Creatine kinase myocardial band
CFU: Colony forming units
C-HD: Cooled hemodialysis
CI: Confidence interval
CVD: Cardiovascular disease
CO: Cardiac output
DSI: Dialysis Symptom Index
DBP: Diastolic blood pressure
eGFR: Estimated glomerular filtration rate
EV: Extracellular vesicles
EDTA: Ethylenediaminetetraacetic acid
EU: Endotoxin units
ECC: Extracorporeal circuit
FGF23 C-term: Fibroblast growth factor 23 C-terminal
GLS: Global longitudinal deformation
HR: Heart rate
HD: Hemodialysis
HDF: Hemodiafiltration
HCT: Hematocrit
Hb: Hemoglobin
HV-HDF: High-volume hemodiafiltration
hs-CRP: High sensitive C-reactive protein
HOLLANT: Effect of various dialysis modalities on intradialytic hemodynamics and cardiac function in chronic dialysis patients
IDH: Intradialytic hypotension
IS: Interspace region
IDWG: Interdialytic weight gain
IL-6R: Interleukin-6 receptor
IS-pro: 16S–23S interspace
KDOQI: Kidney Disease Outcomes Quality Initiative
LV: Left ventricular
LV-HDF: Low-volume hemodiafiltration
LAVI: Left atrial volume index
LMWH: Low-molecular weight heparin
MAP: Mean arterial pressure
MMW: Middle molecular weight
mDNA: Microbial DNA
PTH: Parathyroid hormone
PP: Pulse pressure
PBS: Phosphate-buffered saline
PCR: Polymerase chain reaction
PROMs: Patient reported outcome measures
RV: Right ventricular
RBV: Relative blood volume
RCT: Randomized controlled trial
SPAP: Systolic pulmonary artery pressure
SD: Standard deviation
SBP: Systolic blood pressure
S-HD: Standard hemodialysis
sCD14: Soluble CD14
sv-ICAM-1: Soluble vascular intercellular cell adhesion molecule 1
spO2: Oxygen saturation
SV: Stroke volume
S5–1: Sampled matrix array transducer
STE: Speckle tracking echocardiography
spKt/Vurea: Dialysis single-pool Kt/V for urea
TAPSE: Tricuspid annular plane systolic excursion
Td: Dialysate temperature
TRPS: Tunable resistive pulse sensing
UF: Ultrafiltration
UP: Ultrapure
VAS-TP: Visual Analogue Scale Thermal Perception
